# The Role of Anatomical Imaging and Intraoperative Neuromonitoring (IONM) for Successful Prediction of a Nonrecurrent Laryngeal Nerve

**DOI:** 10.1155/2022/3147824

**Published:** 2022-02-21

**Authors:** Hiroki Kuwazoe, Keisuke Enomoto, Daichi Murakami, Naoko Kumashiro, Saori Takeda, Muneki Hotomi

**Affiliations:** Department of Otolaryngology-Head and Neck Surgery, Wakayama Medical University, Wakayama, Japan

## Abstract

A nonrecurrent laryngeal nerve (NRLN) is a rare anatomical variant of laryngeal nerves that branches directly from the vagus nerve. The anatomical abnormality makes it difficult to identify the NRLN and results in high incidence of accidental nerve injury during surgery. A 76-year-old woman complained of swelling in the right side of her neck and visited our university hospital for further examination. Ultrasonography showed a right thyroid lobe mass with calcification and fine needle aspiration biopsy was classified as class III. Computed tomography revealed that the right subclavian artery branched directly from the descending aorta without branching from the brachiocephalic artery and ran behind the esophagus. Since it was afraid that the accidental injury of NRLN was likely to occur, a right thyroid lobe dissection using intraoperative neuromonitoring (IONM) was performed. After separating the connective tissue on the thyroid capsule from the right side of the trachea to the inferior pole laterally, the NRLN running across the level of the inferior margin of the cricoid cartilage was identified by using IONM 0.5 mA stimulation. After complete dissection of right thyroid lobe, we again stimulated the NRLN by 0.5 mA and the electromyographic response was confirmed. The pathological analysis confirmed nodular hyperplasia without malignancy; the condition was diagnosed as an adenomatous goiter. There was no vocal cord dysfunction and hoarseness after the surgery. IONM contributed to the prevention of NRLN injury during the surgery. We believe that it is important to confirm the presence or absence of an aberrant subclavian artery on preoperative imaging, and that IONM should be considered to identify the NRLN to prevent vocal cord paralysis if its presence is suspected.

## 1. Introduction

A nonrecurrent laryngeal nerve (NRLN) branches directly from the vagus nerve to the larynx, without recurring via the right subclavian artery or the aortic arch. The NRLN is a rare anatomical variant of laryngeal nerves; its reported incidence is from 0.52% to 1.94% [1, 2]. The anatomical abnormality makes it difficult to identify the NRLN and results in high incidence of accidental nerve injury during surgery [3, 4]. Recent surgical adjuncts, such as intraoperative neuromonitoring (IONM), are commonly applied to identify the recurrent laryngeal nerve (RLN) and prevent it injury in thyroid surgery. According to systematic review, to use IONM during thyroid surgery reduces overall and transient RLN palsy incidence ratio but does not contribute to improve persistent RLN palsy ratio [5].

Herein, we report a case in which an NRLN was found in a patient with adenomatous goiter, which was suspected from preoperative imaging findings, and IONM contributed in preventing NRLN injury during the surgery.

## 2. Case Report

The patient was a 76-year-old woman who complained of swelling in the right side of her neck. A general physician measured the mass visible near the right thyroid lobe and noted it to be more than 50 mm. The patient then visited our university hospital for further examination.

**Figure 1:**
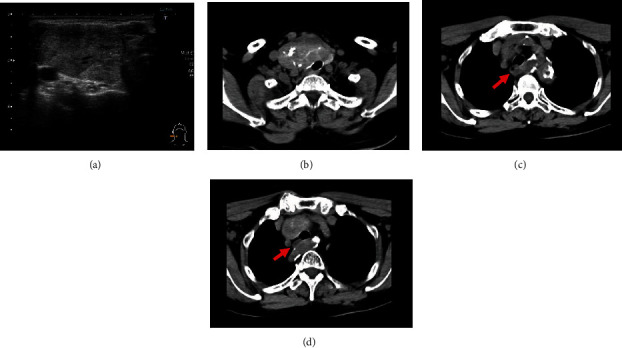
Preoperative imaging. (a) Ultrasonography showing a mass in the right thyroid lobe. (b) Computed tomography image showing the thyroid tumor with calcification compressing the cervical trachea. (c, d) The right aberrant subclavian artery (arrow) running behind the esophagus (^∗^).

Physical examination revealed that the mass in the right thyroid lobe was elastic, hard, and measured approximately 30 mm × 50 mm. Blood examination results were normal, i.e., the levels of thyroid stimulating hormone, free thyroid hormone, and thyroid antibodies were with the normal limits except for thyroglobulin (982.2 ng/mL). Ultrasonography (US) showed a 37 mm × 26 mm × 57 mm mass in the right thyroid lobe with petechial calcification and coarse central calcification (Figure 1(a)). Cervicothoracic plain computed tomography (CT) showed that the right thyroid lobe mass with calcification was compressing the cervical trachea (Figure 1(b)). Additionally, the right subclavian artery branched directly from the descending aorta without branching from the brachiocephalic artery and ran behind the esophagus (Figures 1(c) and 1(d)).

Upon fine needle aspiration cytology examination, the right thyroid lobe mass was classified as class III. There were no obvious intranuclear cytoplasmic inclusion bodies or nuclear grooves; no obvious papillary carcinoma was observed. The primary cell origin was considered to be the follicular epithelium.

**Figure 2:**
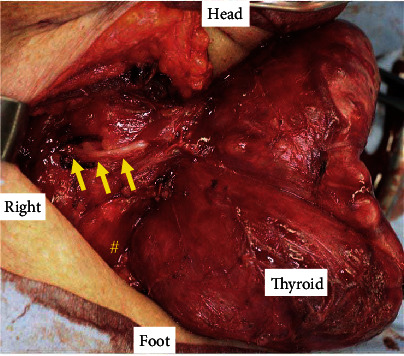
Intraoperative photographs. Nonrecurrent laryngeal nerve (arrows) branching directly from the vagus nerve. ^#^Cervical esophagus.

Right lobectomy of the thyroid was performed to remove the mass compressing the trachea. A collar incision was made at the central level of the anterior neck mass, and we approached the right thyroid lobe lateral to the avascular area. Initially, the common carotid artery was identified, and the thyroid was dissected towards the inferior pole in shallow layers. After separating the connective tissue on the thyroid capsule from the right side of the trachea to the inferior pole laterally, a nerve running across the level of the inferior margin of the cricoid cartilage was identified by using IONM 0.5 mA stimulation (Medtronic, Jacksonville, FL). This nerve from vagus nerve to larynx was exposed, and it was confirmed as right NRLN (Figure 2). The right thyroid lobe was resected, and then, we again stimulated the NRLN by 0.5 mA, and the electromyographic response was confirmed.

Pathological analysis confirmed nodular hyperplasia without malignancy; it was diagnosed as an adenomatous goiter. Postoperative laryngeal endoscopy showed that the mobility of the vocal cord was good; there was no vocal cord dysfunction and hoarseness after the surgery. There were no postoperative complications, and the patient was discharged on the fifth postoperative day. Written informed consent was obtained from the patient for publication of the case report and the accompanying images.

## 3. Discussion

Aberrant right subclavian artery is one of the most common embryologic abnormalities. When arteria lusoria is present, the brachiocephalic trunk is absent and the aberrant branch arises as the fourth branch from the distal left aortic arch and coursing rightwards behind the esophagus. NRLN is strongly associated with arteria lusoria and increases risk of iatrogenic RLN injury [6].

Postoperative vocal cord paralysis is the most frequently problematic postoperative complication for patients undergoing thyroid surgery. It causes hoarseness and, sometimes, serious dysphagia. It is known that vocal cord paralysis occurs in 3% to 6% patients who underwent surgery for benign thyroid diseases [7, 8]. While approximately 90% of patients recover from vocal cord palsy within 12 months after the surgery, the remaining 10% of patients suffer through this as a permanent complication [7]. Toniato et al. stated that the incidence of postoperative vocal cord paralysis was 1.8% in patients with normal recurrent laryngeal nerve, while the incidence was 12.9% in patients with NRLN [3]. The higher incidence of vocal cord paralysis in the NRLN group may be due to the difficulty of identification of the nerve because of its aberrant location. In the present case, we could identify the NRLN using the novel IONM [9, 10], and were able to avoid injury that may cause vocal cord paralysis. Donatini et al. also showed the decreased incidence of nerve palsy, especially in case of NRLN, by applying IONM for surgery [11].

CT and US images are reported to be useful for the preoperative diagnosis of NRLN [2, 12–14]. The detection of NRLN by CT is excellent; Satoh et al. reported that the sensitivity and specificity of CT for detection of right-sided NRLN were 100% and 100%, respectively [12]. Contrastingly, the sensitivity and specificity of cervical US for detection of right NRLN were 100% and 83.5%, respectively. Other studies also reported that the sensitivity and specificity varied between 99% to 100% and 41% to 100% in 3,740 patients who underwent neck US for the detection of NRLN [2]. Although CT has higher specificity for detecting NRLN, US can be used preferentially to identify NRLN based on its high sensitivity, cost, and radiation exposure. However, US has some limitation to identify NRLN. In this case, US was not able to detect the bifurcation of the brachiocephalic artery due to the artifacts of the thyroid mass and the clavicle. The clinical importance of contrast-enhanced CT and three-dimensional reconstruction for detecting aberrant subclavian artery is well known [15, 16]. Although contrast-enhanced CT was not performed because of moderate renal dysfunction in our case, we could easily identify the right aberrant subclavian artery even with plain imaging.

IONM has been widely used for the identification and monitoring of RLN in thyroid and parathyroid surgery. Intraoperative neuromonitoring guidelines in thyroid and parathyroid surgery have been published in 2011 from International IONM Study Group, and the standard procedure for performing IONM has been outlined [10]. They suggested a four-step procedure as follows: (a) the vagus nerve identification in the neurovascular bundle and touch by the stimulator probe, after medial traction of the thyroid lobe, V1; (b) the RLN stimulation when first identified, R1; (c) the second RLN stimulation, after complete dissection of the lateral thyroid lobe, R2; and (d) the vagus nerve stimulation after complete dissection, V2. In this case, we followed the standard clinical protocol of IONM in Japan [17], and we omit V1 and V2 steps.

In summary, we encountered a case in which NRLN was predicted by preoperative CT scan and preserved it by using IONM. It is important to confirm the presence or absence of an aberrant subclavian artery on preoperative imaging, and that IONM should be considered to identify the NRLN to prevent vocal cord paralysis if its presence is suspected.
